# Knockdown of circSMAD2 inhibits the tumorigenesis of gallbladder cancer through binding with eIF4A3

**DOI:** 10.1186/s12885-021-08895-1

**Published:** 2021-11-02

**Authors:** Yiyu Qin, Yongliang Zheng, Cheng Huang, Yuanyuan Li, Min Gu, Qin Wu

**Affiliations:** 1 Clinical Medical College, Jiangsu Vocational College of Medicine, 283 Jiefang South Road, Yancheng, 224005 Jiangsu China; 2 Rehabilitation College, Jiangsu Vocational College of Medicine, Yancheng, 224005 Jiangsu China

**Keywords:** circSMAD2, SMAD2, eIF4A3

## Abstract

**Background:**

Gallbladder cancer (GBC) is the seventh most common gastrointestinal cancer worldwide. This study aimed to investigate the function of circSMAD2 in GBC.

**Methods:**

To investigate the function of circSMAD2 in GBC, the level of circSMAD2 in GBC cells was detected by RT-qPCR. CCK-8 assay was performed to investigate the cell viability. Cell apoptosis was tested by flow cytometry. In addition, transwell assay was used to detect the cell migration and invasion. RIP and RNA pull-down were used to explore the relation among circSMAD2, eIF4A3 and SMAD2. Meanwhile, xenograft mice model was established to investigate the function of circSMAD2 in GBC.

**Results:**

The data revealed that circSMAD2 was upregulated in GBC, and circSMAD2 knockdown significantly inhibited the viability of GBC cells. In addition, circSMAD2 siRNA notably induced the apoptosis in GBC cells. The migration and invasion of GBC cells were obviously suppressed in the presence of circSMAD2 siRNA. Meanwhile, circSMAD2 suppressed the binding between eukaryotic translation initiation factor 4A3 (eIF4A3) and SMAD2 through binding with eIF4A3. Knockdown of circSMAD2 notably inhibited the expression of SMAD2 in GBC cells, and SMAD2 overexpression partially reversed the anti-tumor effect of circSMAD2 knockdown. Finally, circSMAD2 siRNA significantly inhibited the tumor growth of GBC in vivo.

**Conclusion:**

Knockdown of circSMAD2 inhibits the tumorigenesis of gallbladder cancer through binding with eIF4A3. Thus, our study provided a new strategy for the treatment of GBC.

**Supplementary Information:**

The online version contains supplementary material available at 10.1186/s12885-021-08895-1.

## Introduction

Gallbladder cancer (GBC) is the seventh most common gastrointestinal cancer worldwide, and high prevalence rates of this aggressive disease have been observed in Chile, India and Japan [1, 2]. Patients with GBC are usually diagnosed at advanced stage due to the lack of specific symptoms in early stages [3]. Nowadays, the major treatments of GBC are surgery and chemotherapy, while the effect remains not ideal [4]. Great efforts have been made to study GBC; however, the outcomes remain limited. Thereby, it is urgent to find new therapeutic targets for the treatment of GBC.

Circular RNAs (CircRNAs) are a group of endogenous RNAs which have a stable loop structure [5, 6]. CircRNAs play important roles in cell biological functions such as gene expression, post-transcriptional modification and protein synthesis [7–9]. Moreover, the dysregulation of circRNAs is known to be closely associated with the progression of GBC. For instance, Huang X et al. found that circERBB2 could contribute to the progression of GBC through mediation of PA2G4-dependent rDNA transcription [10]; Wang S et al. indicated that circFOXP1 could promote the tumorigenesis of GBC via regulation of PKLR [11]. Meanwhile, it has been reported that circSMAD2 could regulate the tumorigenesis of prostate cancer and hepatocellular carcinoma [12, 13]. However, the role of circSMAD2 in GBC remains unclear.

In this study, we sought to investigate the function of circSMAD2 in GBC, we hope this research would shed new lights on exploring the new strategies for the treatment of GBC.

## Material and methods

### Cell culture

GBC-SD cells were obtained from Cell Bank of the Chinese Academy of Science (Shanghai, China), and G415 cell lines were purchased from RIKEN Cell Engineering Division-Cell Bank (Tokyo, Japan). Human intrahepatic bile duct cell lines (HIBEpiC) were purchased from American Type Culture Collection (ATCC). Cells were maintained in Dulbecco’s Modified Eagle’s Medium (DMEM, Thermo Fisher Scientific) with 10% FBS (Thermo Fischer Scientific), 1% penicillin and streptomycin (Thermo Fisher Scientific) in the condition with 37 °C, 5% CO_2_.

### Quantitative real time polymerase chain reaction (RT-qPCR)

TRIzol reagent (TaKaRa, Tokyo, Japan) was used to extract total RNA from cell lines or tissues according to the manufacturer’s protocol. Reverse transcription kit (TaKaRa, Ver.3.0) was used to synthesize cDNA. RT-qPCRs were performed in triplicate under the following protocol: 2 min at 94 °C, followed by 35 cycles (30 s at 94 °C and 45 s at 55 °C). The primers were purchased from GeneCreate Biological Engineering Co., Ltd. (Wuhan, China). CircSMAD2: forward, 5′-ATGGACACAGGTTCGATA-3′ and reverse 5′-CAACTGGCGGCGTGAATG-3′. SMAD2: forward, 5′-CTTTGTGCAGAGCCCCAATT-3′ and reverse 5′-CTTGTTACCGTCTGCCTTC-3′. β-actin: forward, 5′-GTCCACCGCAAATGCTTCTA-3′ and reverse 5′-TGCTGTCACCTTCACCGTTC-3′. 2^-ΔΔCt^ method was used to quantify the data. Meanwhile, the internal reference gene (β-actin) was used for normalization.

### Cell transfection

circSMAD2 siRNAs (circSMAD2 siRNA1, circSMAD2 siRNA2 and circSMAD2 siRNA3), eiF4A3 siRNA or negative control RNAs (all at 10 nM) were transfected into GBC cells using Lipofectamine 2000 (Invitrogen, Carlsbad, CA). circSMAD2 siRNAs, eiF4A3 siRNA and negative control RNAs were purchased from GenePharma (Shanghai, China).

For SMAD2 overexpression, GBC cells were transfected with pcDNA3.1 or pcDNA3.1-SMAD2 (SMAD2 oE) by using Lipofectamine 2000 (Invitrogen). pcDNA3.1 and pcDNA3.1-SMAD2 were obtained from Genepharma.

### Tissue collection

In total, 20 pairs of GBC samples and adjacent normal tissues were collected from clinical medical college, Jiangsu Vocational College of Medicine between April 2019 and April 2020. The patients were informed of the purpose of the experiments and provided written informed consent. Meanwhile, the samples were used for investigation of circSMAD2 levels. The present study was approved by the Institutional Ethical Committee of Clinical Medical College, Jiangsu Vocational College of Medicine (approval no. 20200414027). In addition, all methods involving human tissues were performed in accordance with the relevant guidelines and regulations. Moreover, The clinical feature of patients with GBC was provided in Table 1. The Samples selection criteria were as follow: collected from the people (≥18 years old) who have been diagnosed with GBC and have undergone surgery without chemotherapy.

**Table 1:** Association between circSMAD2 expression and clinicopathologic characteristics of GBC patients

Characteristics	Cases	Low expression of circSMAD2 (%)	High expression of circSMAD2 (%)	χ^**2**^ value	***P*** value
Sex				0.833	0.3613
Male	8	5 (50%)	3 (42.9%)		
Female	12	5 (50%)	7 (57.1%)		
Age (Years)				0.800	0.3711
< 60	10	6 (60%)	4 (40%)		
≥ 60	10	4 (40%)	6 (60%)		
TNM stage (AJCC)				1.978	0.1596
0-II	7	5 (50%)	2 (20%)		
III-IV	13	5 (50%)	8 (80%)		

### CCK-8 assay

GBC cells (5 × 10^3^ per well) were seeded overnight. Then, cells were treated with negative control (si-ctrl), circSMAD2 siRNA1 or circSMAD2 siRNA3 for 48 h. 10 μl CCK-8 reagent (Beyotime, Shanghai, China) were added to each well and further incubated for 2 h at 37 °C. Finally, the absorbance of GBC cells was measured at 450 nm using a microplate reader (Thermo Fisher Scientific).

### Cell apoptosis analysis

G415 or GBC-SD cells were trypsinized, washed with PBS and resuspended in Annexin V Binding Buffer, followed by staining with 5 μl FITC and 5 μl propidium (PI) in the dark for 15 min. Cells were analyzed using flow cytometer (BD, Franklin Lake, NJ, USA) to test the cell apoptosis rate.

### RNA-binding protein immunoprecipitation (RIP)

The correlation between circSMAD2 (or SMAD2) and eIF4A3 was investigated by RIP assay. In brief, anti-eIF4A3 antibody (Millipore) was used to immunoprecipitate the chromatin, and immunoglobulin G (IgG) was performed as negative control. Lastly, the enrichment of circSMAD2 or SMAD2 was tested using RT-qPCR. The procedure was in accordance with the previous reference [14].

### Transwell assay

The upper chambers were pretreated with 50 μl Matrigel (BD) (Matrigel was not involved in migration assay). Then, GBC cells (1 × 10^6^ cells/ml) were seeded into the upper chambers (the medium did not contain FBS). The lower chambers were supplemented with RPMI 1640 medium (1% FBS). Cells which attached to the underside of the membrane were fixed and stained with a 0.1% crystal violet after 24 h of incubation. After that, the migratory or invaded cells were observed under a microscope (400x magnification).

### Western-blot detection

GBC cells or tissues were isolated from cells by RIPA buffer and quantified by BCA kit (Beyotime). Proteins were separated with SDS-PAGE (10%), and then proteins were transferred onto PVDF (Bio-Rad) membranes. After that, the membranes were incubated with primary antibodies at 4 °C overnight after blocked with 5% skim milk for 1 h. The primary antibodies were as follows: anti-SMAD2 (Abcam, Cambridge, MA, USA; 1:1000), anti-cleaved caspase 3 (Abcam; 1:1000) and anti-β-actin (Abcam; 1:1000). Then, the membranes were incubated with secondary antibody (HRP-conjugated goat anti-rabbit IgG; Abcam, 1:5000) at room temperature for 1 h. β-actin was used as an internal control. Enhanced chemiluminescence reagent (Thermo Fisher Scientific, Inc.) was used to visualize the protein bands. ImageJ software (version 2.0; National Institutes of Health) was used to quantify the intensity of the bands.

### RNA pull-down

For the RNA pulldown assay, secondary structure formation was induced by RNA structure buffer (ThermoFisher). Meanwhile, the biotinylated circSMAD2 (GenePharma) and bio-NC were coated to magnetic beads. Subsequently, Cells were incubated with the magnetic beads. After 6 h of incubation, the RNA was extracted and PCR was performed to detect the enrichment of eIF4A3.

### In vivo experiments

Twelve BALB/c nude mice (6–8 weeks old) were purchased from Vital River (Beijing, China). GBC cells were subcutaneously transplanted into mice according to the previous reference [15]. Vector-control or circSMAD2 siRNA (10 nM) was injected intra-tumor twice weekly when the tumor volume reached about 200 mm^3^. The tumor volume was investigated once a week according to the formula: Length×Width×Width/2. At the end of the experiments, mice were sacrificed and the tumors were collected and weighted. All in vivo experiments were performed in accordance with National Institutes of Health guide for the care and use of laboratory animals, following a protocol approved by the Ethics Committees of Clinical Medical College, Jiangsu Vocational College of Medicine (No. 20200526032).

### Statistical analysis

Each group were performed at least three independent experiments and all data were expressed as the mean ± standard deviation (SD). Differences were analyzed using Student’s t-test (only 2 groups) or one-way analysis of variance (ANOVA) followed by Tukey’s test (more than 2 groups, Graphpad Prism7). *P* < 0.05 was considered to indicate a statistically significant difference.

## Results

### Knockdown of circSMAD2 significantly inhibited the viability of GBC cells

**Fig. 1:**
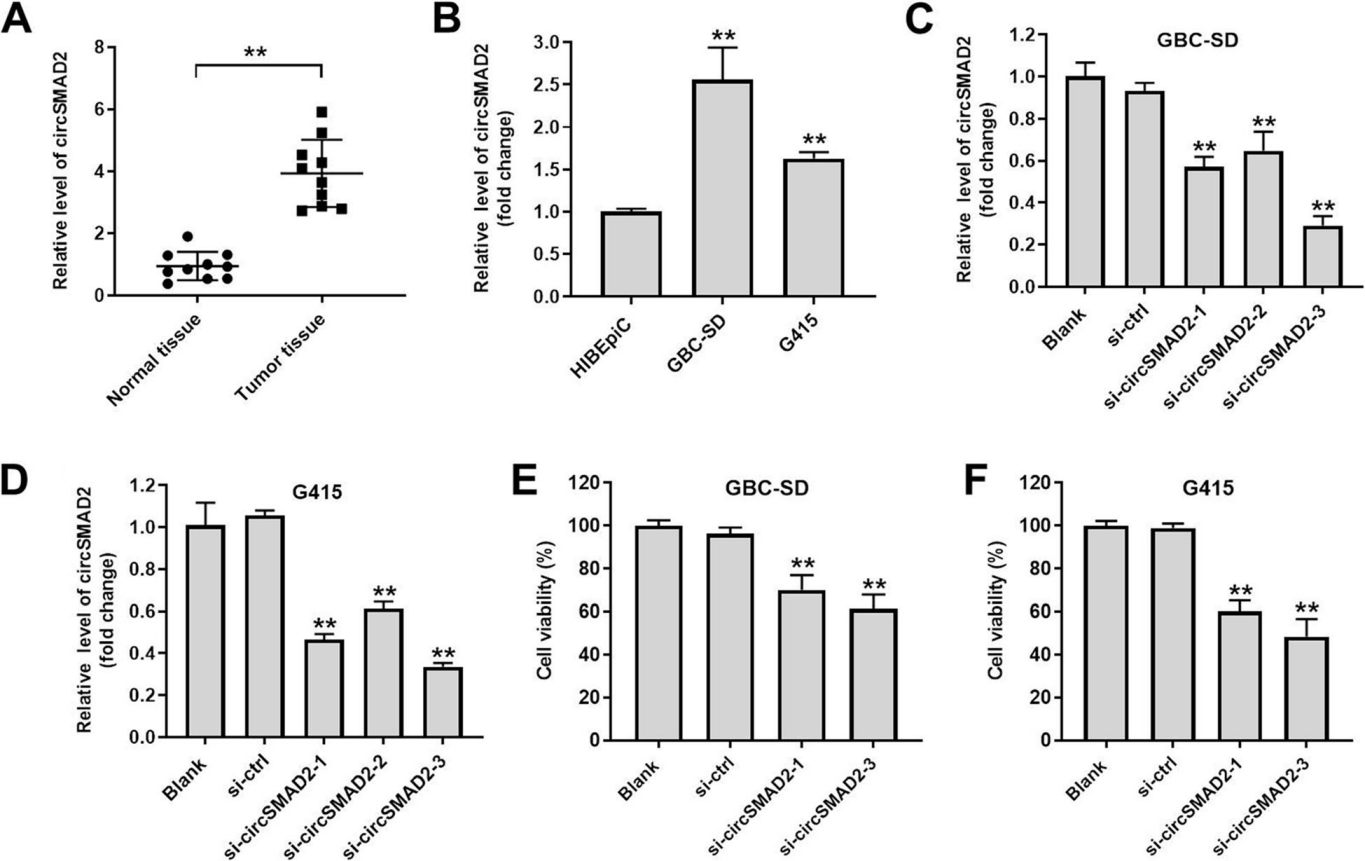
Knockdown of circSMAD2 significantly inhibited the viability of GBC cells. **(A)** The expression of circSMAD2 in GBC and adjacent normal tissues was detected by RT-qPCR. **(B)** The level of circSMAD2 in HIBEpic, GBS-SD and G415 cells was investigated by RT-qPCR. **(C, D)** GBC cells were transfected with circSMAD2 siRNA1, circSMAD2 siRNA2 or circSMAD2 siRNA3. Then, the efficiency of cell transfection was tested by RT-qPCR. **(E, F)** GBC cells were transfected with circSMAD2 siRNA1 or circSMAD2 siRNA3. The viability of GBC cells was investigated by CCK-8 assay. ^**^P < 0.01 compared to normal tissues, HIBEpic or blank

To investigate the role of circSMAD2 in GBC, RT-qPCR was performed. As indicated in Fig. 1A, the expression of circSMAD2 in GBC tissues was significantly higher, compared with adjacent normal tissues. Consistently, the level of circSMAD2 in GBC cells was notably higher than that in HIBEpic cells (Fig. 1B). CircSMAD2 level was not correlated with sex, age and TNM stage of patients with GBC (Table 1). Meanwhile, the expression of circSMAD2 in GBC cells was significantly decreased by circSMAD2 siRNAs (Fig. 1C and D). Moreover, the viability of GBC cells was notably inhibited by circSMAD2 knockdown (Fig. 1E and F). Altogether, knockdown of circSMAD2 significantly inhibited the viability of GBC cells. Since GBC cells were more susceptible to si-circSMAD2–3, compared with si-circSMAD2–1, si-circSMAD2–3 were selected of use in subsequent experiments.

### Silencing of circSMAD2 notably induced the apoptosis and inhibited the migration and invasion of GBC cells

**Fig. 2:**
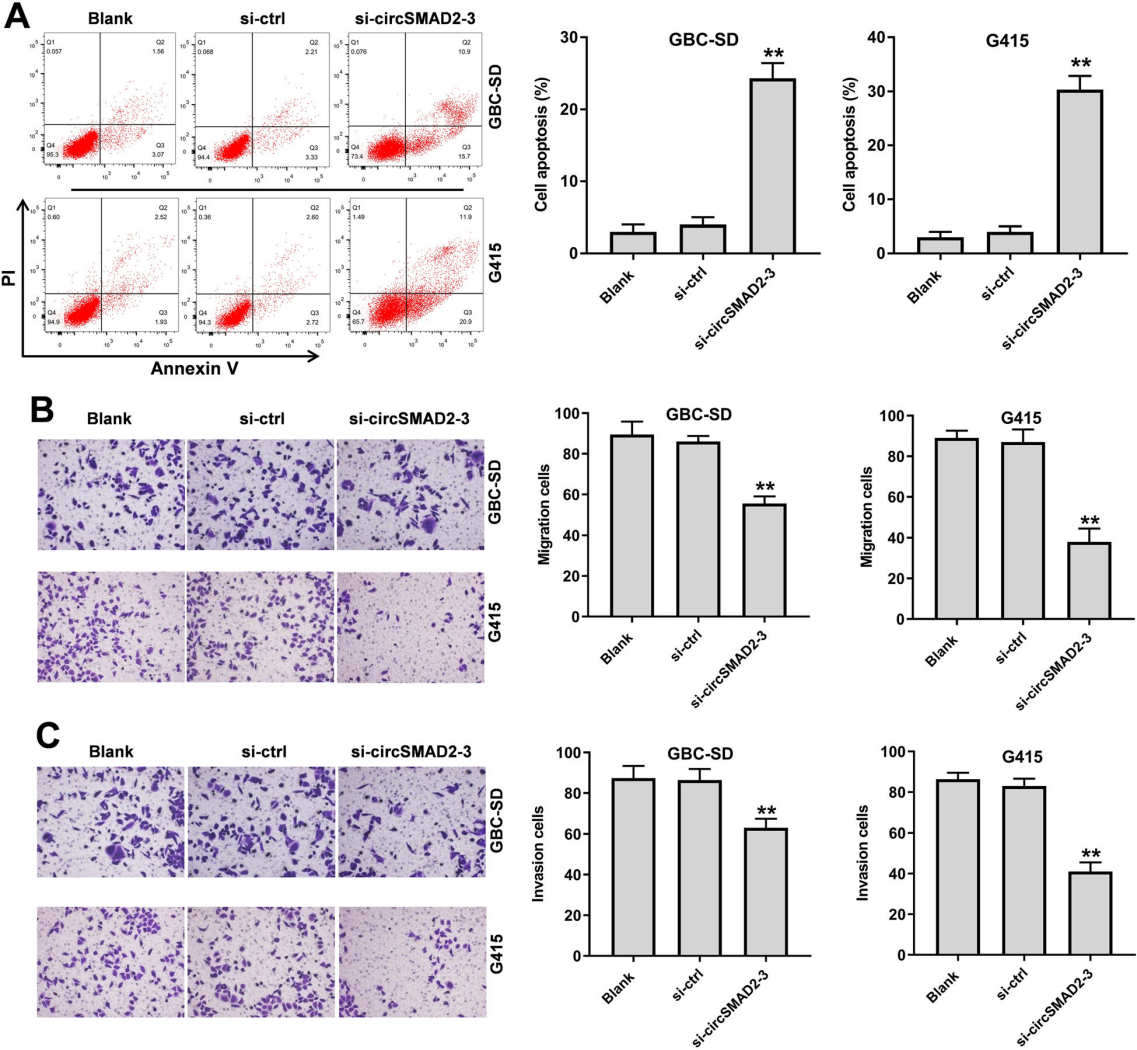
Silencing of circSMAD2 notably induced the apoptosis and inhibited the migration and invasion of GBC cells. **(A)** The apoptosis of GBC cells was tested by flow cytometry. **(B)** The migration of GBC cells was investigated by transwell assay. **(C)** The invasion of GBC cells was investigated by transwell assay. ^**^P < 0.01 compared to blank

In order to detect the function of circSMAD2 in GBC, flow cytometry, migration and invasion assays were used. The data revealed that circSMAD2 siRNA markedly induced the apoptosis in GBC cells (Fig. 2A). In addition, the migration and invasion of GBC cells were significantly decreased in the presence of circSMAD2 siRNA (Fig. 2B and C). Taken together, silencing of circSMAD2 notably induced the apoptosis and inhibited the migration and invasion of GBC cells.

### Knockdown of circSMAD2 inhibited the expression of SMAD2 in GBC cells

**Fig. 3:**
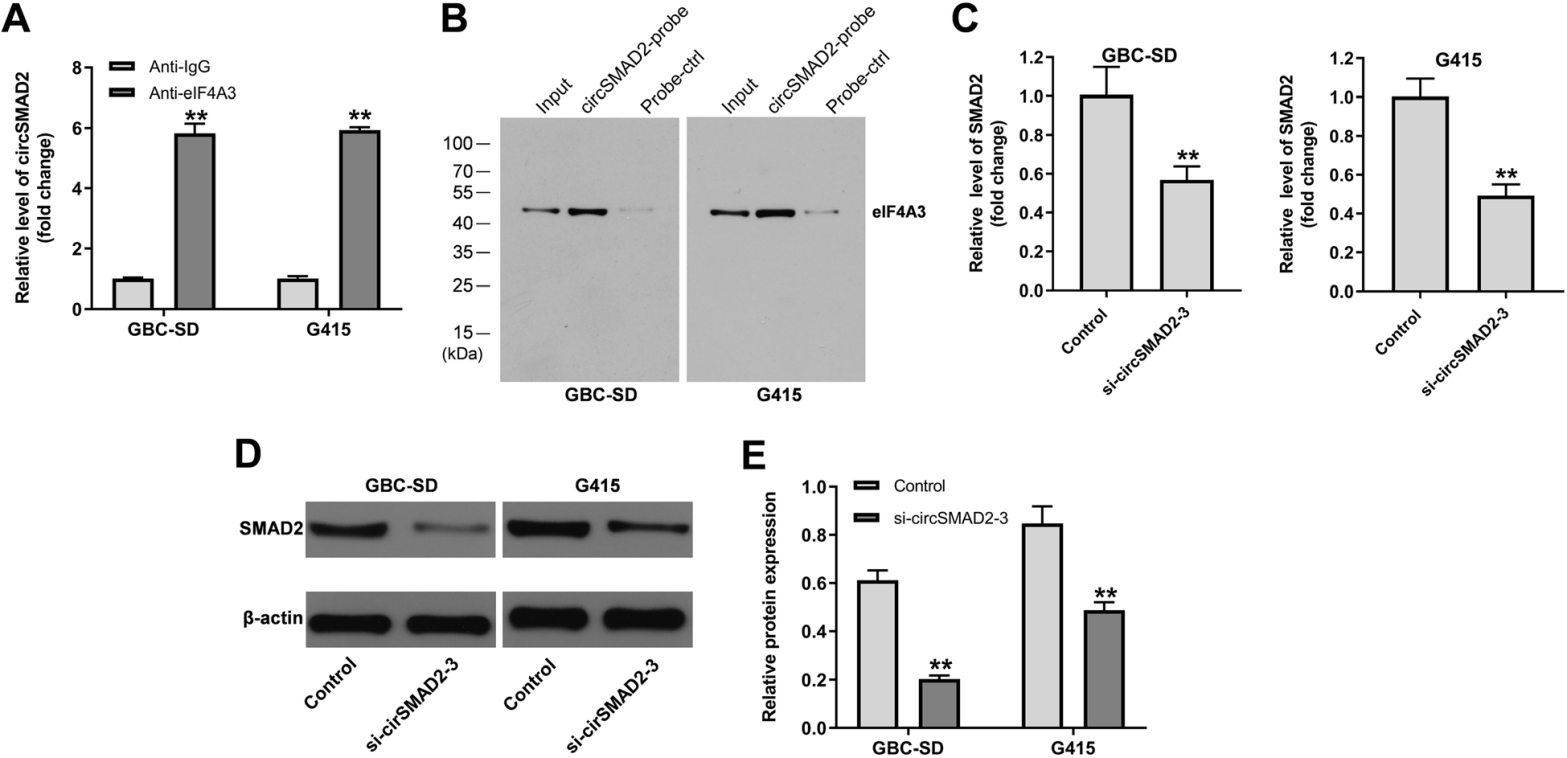
Knockdown of circSMAD2 inhibited the expression of SMAD2 in GBC cells. **(A)** The enrichment of circSMAD2 was tested by RIP. **(B)** The correlation between circSMAD2 and eIF4A3 was explored by RNA pull-down. **(C)** The level of SMAD2 in GBC cells was detected by RT-qPCR. **(D, E)** The protein level of SMAD2 in GBC cells was investigated by western blot. The relative level was quantified by normalizing to β-actin. ^**^P < 0.01 compared to control

In order to investigate the correlation between circSMAD2 and eIF4A3 in GBC, RIP was used. As shown in Fig. 3A, the relative level of circSMAD2 in GBC cells was significantly upregulated by anti-eIF4A3. Consistently, the data of RNA pull-down revealed that circSMAD2 bound with eIF4A3 (Fig. 3B). In addition, the expression of SMAD2 in GBC cells was notably decreased in the presence of circSMAD2 knockdown (Fig. 3C-3E). To sum up, knockdown of circSMAD2 inhibited the expression of SMAD2 in GBC cells.

### SMAD2 bound with eIF4A3

**Fig. 4:**
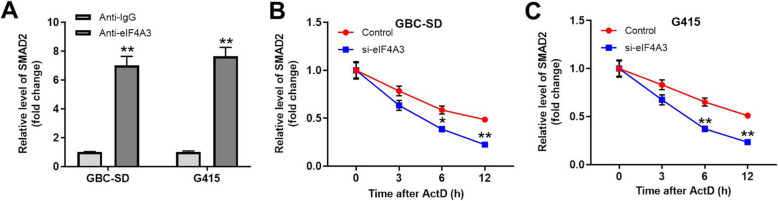
CircSMAD2 bound with eIF4A3. **(A)** The enrichment of SMAD2 was tested by RIP. **(B, C)** The mRNA stability of SMAD2 in actinomycin D-treated GBC cells was analyzed by RT-qPCR. ^**^P < 0.01 compared to control

To explore the relation between SMAD2 and eIF4A3, RIP was used. As indicated in Fig. 4A, the enrichment of SMAD2 in GBC cells was significantly upregulated by anti-eIF4A3. Moreover, knockdown of eIF4A3 could significantly inhibit the mRNA stability of SMAD2 in GBC cells (Fig. 4B and C). In summary, SMAD2 bound with eIF4A3.

### CircSMAD2 knockdown inhibited the growth of GBC cells through regulation of SMAD2

**Fig. 5:**
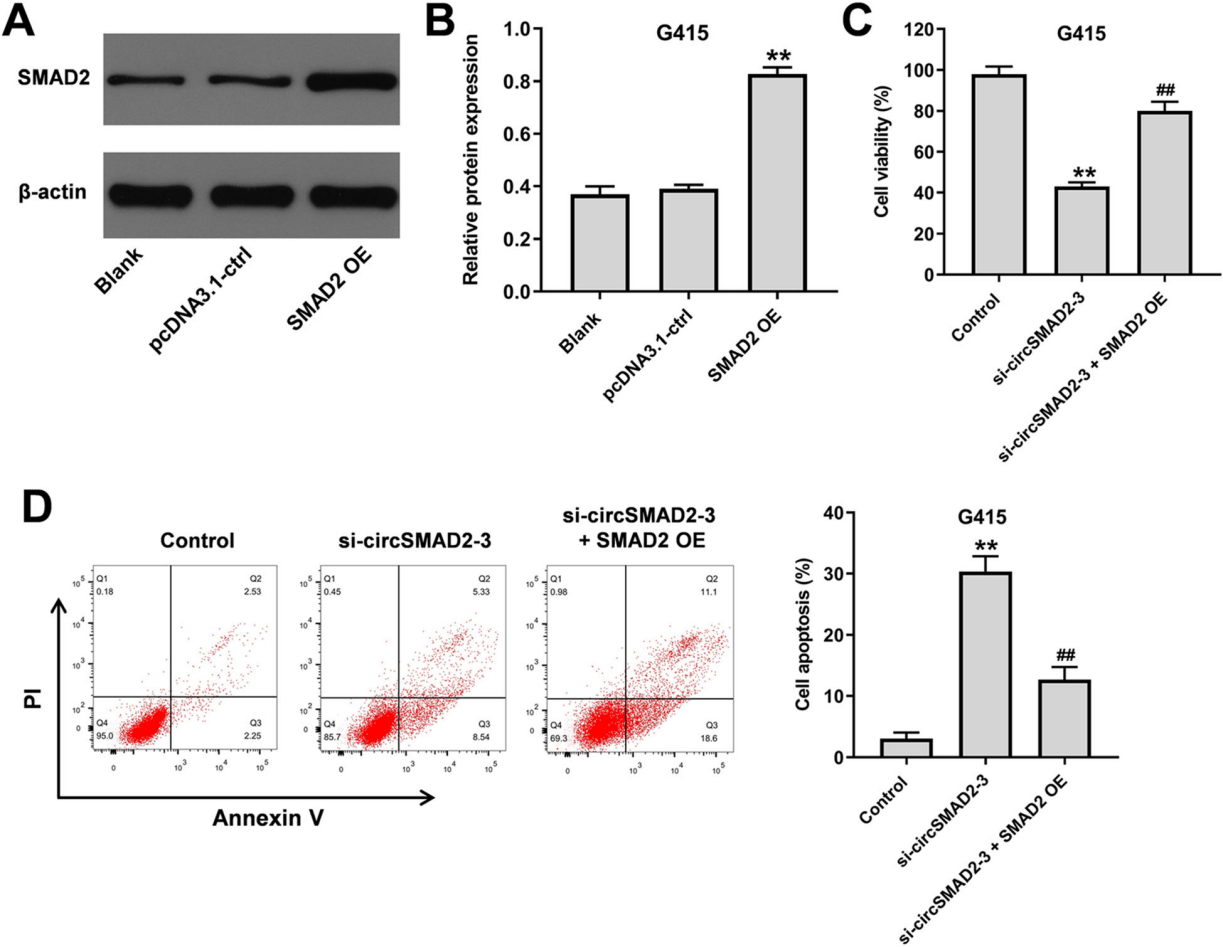
CircSMAD2 knockdown inhibited the growth of GBC cells through regulation of SMAD2. **(A, B)** GBC cells were transfected with pcDNA3.1-SMAD2. Then, the efficiency of cell transfection was investigated by western blot. **(C)** The viability of GBC cells was tested by CCK-8 assay. **(D)** The apoptosis of GBC cells was tested by flow cytometry. ^**^P < 0.01 compared to control. ^##^P < 0.01 compared to si-circSMAD2–3

To further confirm the relation between circSMAD2 and SMAD2 in GBC cells, cells were transfected with pcDNA3.1-SMAD2, and then western blot was used to detect the efficiency of cell transfection. As revealed in Fig. 5A and B, the level of SMAD2 in GBC cells was significantly upregulated by pcDNA3.1-SMAD2. In addition, overexpression of SMAD2 notably reversed circSMAD2 siRNA-induced GBC cell growth inhibition (Fig. 5C and D). Taken together, circSMAD2 knockdown inhibited the growth of GBC cells through regulation of SMAD2.

### Knockdown of circSMAD2 significantly inhibited the tumor growth of GBC in vivo

**Fig. 6:**
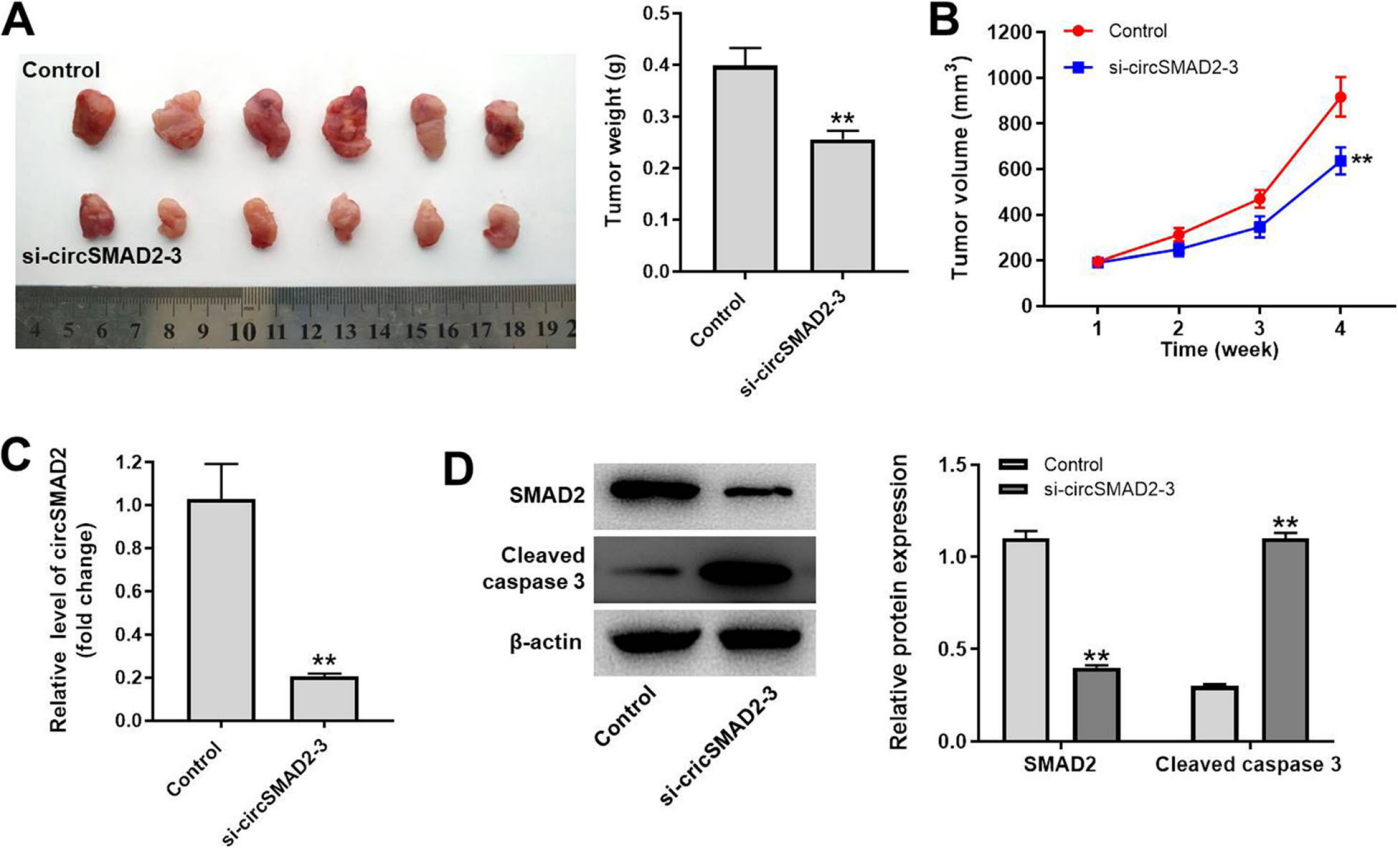
Knockdown of circSMAD2 significantly inhibited the tumor growth of GBC in vivo. **(A)** At the end of study, mice were sacrificed for the collection of tumor tissues, and then tissues were pictured and weighted. **(B)** The volume of tissues was tested weekly. **(C)** The level of circSMAD2 in tumor tissues of mice was detected by RT-qPCR. **(D)** The protein levels of SMAD2 and cleaved caspase 3 in tumor tissues of mice were investigated by western blot. The relative expressions were quantified by normalizing to β-actin. ^**^P < 0.01 compared to control

To further investigate the function of circSMAD2 in GBC, xenograft mice model was established. As shown in Fig. 6A and B, tumor size and weight of mice were significantly reduced by knockdown of circSMAD2. In addition, the level of circSMAD2 in tissues of mice was significantly downregulated in the presence of circSMAD2 siRNA (Fig. 6C and D). In contrast, circSMAD2 siRNA notably increased the expression of cleaved caspase 3 in tissues of mice (Fig. 6D). In summary, knockdown of circSMAD2 significantly inhibited the tumor growth of GBC in vivo.

## Discussion

It has been reported that circSMAD2 knockdown could regulate the tumorigenesis of prostate cancer and hepatocellular carcinoma [16, 17]. In this research, circSMAD2 was upregulated in GBC, and circSMAD2 knockdown could inhibit the growth of GBC cells. Therefore, this study firstly explored the the role of circSMAD2 in GBC.

RBPs are required for modulation of the circRNA biogenesis in a positive or negative way [18, 19]. In this research, our data showed that circSMAD2 could bind with eIF4A3. eIF4A3 is known to be a member of the DEAD box protein family which is involved in nuclear and mitochondrial splicing [20]. Previous studies have revealed that eIF4A3 upregulated the level of circMMP9 and circSEMA5A via binding to pre-mRNAs [21, 22]. Consistently, in our study, we found that eIF4A3 could bind with circSMAD2. In addition, we found a binding site between eIF4A3 and SMAD2. Therefore, it can be concluded that this binding might be responsible for the suppression of SMAD2. In summary, these findings demonstrated that RBPs could participate in the biological function of circRNAs which may lead to diverse regulatory mechanisms. In the future, our research will continue to investigating more RBP-based diverse modulation of circRNA biogenesis.

TGF-β signaling plays a crucial role in various tumors, including GBC [23, 24]. It has been reported that TGF-β can activate SMAD2 [25, 26]. In this study, we found that circSMAD2 knockdown downregulated the expression of SMAD2 in GBC cells. Based on these data, the mechanism underlying the anti-tumor effects of circSMAD2 knockdown was associated with the inactivation of TGF-β signaling pathway. Meanwhile, our current study revealed that knockdown of eIF4A3 could inhibit the transcription of SMAD2. Based on the data, circSMAD2 could inhibit the binding between eIF4A3 and SMAD2 through binding with eIF4A3.

Frankly speaking, there are some shortcomings in this research as follows: 1) the relation between circSMAD2 and eIF4A3 in GBC needs to be further explored; 2) more mechanisms by which circSMAD2 regulate the tumorigenesis of GBC remain to be further explored. Therefore, more investigations are needed in coming future.

In conclusion, knockdown of circSMAD2 inhibits the tumorigenesis of gallbladder cancer through binding with eIF4A3. Thus, our study might shed new lights on exploring the new strategies against GBC.

## Supplementary Information

**Additional file 1.** .

## Data Availability

The datasets used and/or analysed during the current study are available from the corresponding author on reasonable request.
