# Supplementary material for: Knockdown of circSMAD2 inhibits the tumorigenesis of gallbladder cancer through binding with eIF4A3
Source: BMC Cancer. 2021 Nov 2;21:1172. doi: 10.1186/s12885-021-08895-1 (PMC8564960; doi:10.1186/s12885-021-08895-1)

Figure 3D G415 Smad2 Figure 3D GBC-SD Smad2


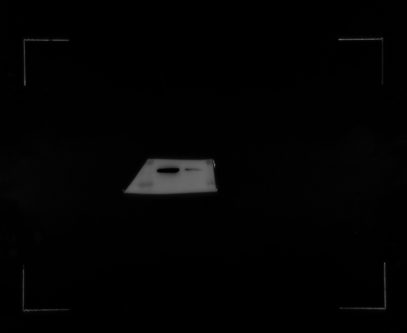

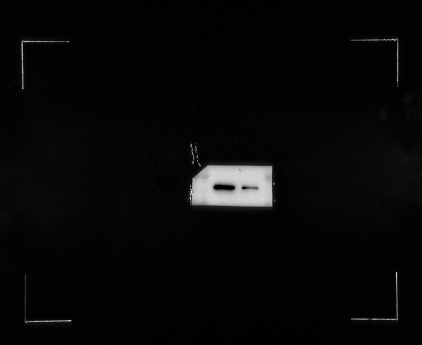


Figure 3D G415 β-actin Figure 3D GBC-SD β-actin


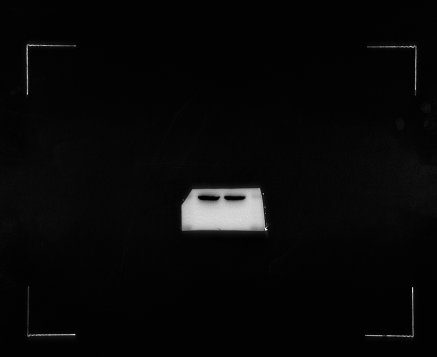

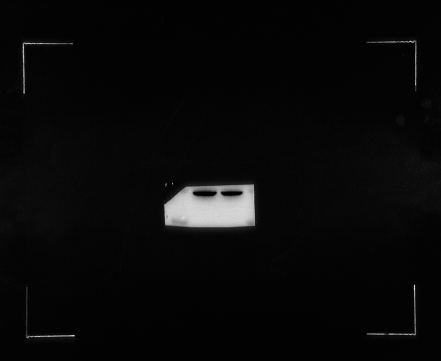


Figure 5A Smad2 Figure 5A β-actin


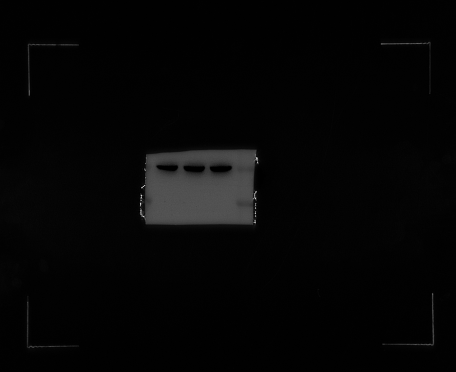


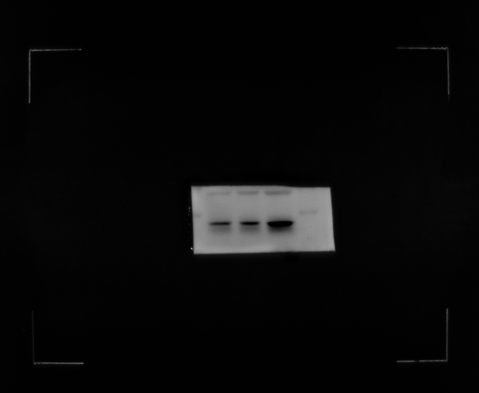


Figure 6D Smad2 Figure 6D cleaved caspase 3


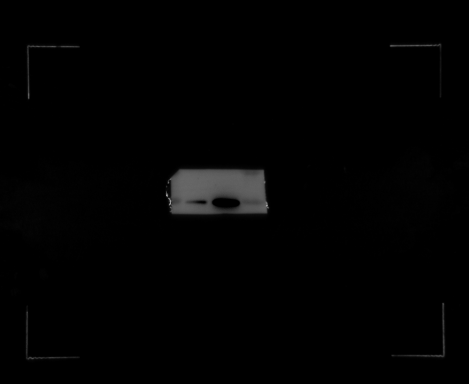

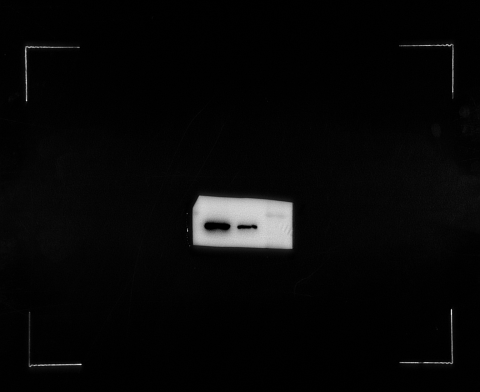


Figure 6D β-actin


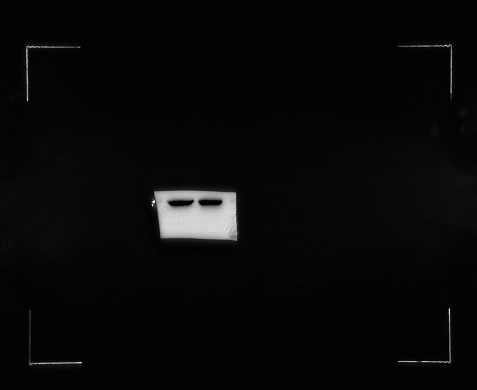

Supplement: Supplementary file 1 — Additional file 1. . [file 12885_2021_8895_MOESM1_ESM.docx]
